# Indirect cholinergic activation slows down pancreatic cancer growth and tumor-associated inflammation

**DOI:** 10.1186/s13046-020-01796-4

**Published:** 2020-12-24

**Authors:** Paulo L. Pfitzinger, Laura Fangmann, Kun Wang, Elke Demir, Engin Gürlevik, Bettina Fleischmann-Mundt, Jennifer Brooks, Jan G. D’Haese, Steffen Teller, Andreas Hecker, Moritz Jesinghaus, Carsten Jäger, Lei Ren, Rouzanna Istvanffy, Florian Kühnel, Helmut Friess, Güralp Onur Ceyhan, Ihsan Ekin Demir

**Affiliations:** 1 Department of Surgery, Klinikum rechts der Isar, Technical University of Munich, School of Medicine, Ismaninger Str. 22, 81675 Munich, Germany; 2 Key laboratory of Carcinogenesis and Translational Research (Ministry of Education), Department of Hepatic, Biliary & Pancreatic Surgery, Peking University School of Oncology, Beijing Cancer Hospital & Institute, Beijing, 100710 China; 3 Department of Gastroenterology, Hepatology, and Endocrinology, Hannover Medical School, Hannover, Germany; 4 Department of General, Visceral, and Transplantation Surgery, Ludwig-Maximilians-University Munich, Munich, Germany; 5 Department of General and Thoracic Surgery, University Hospital of Giessen, Giessen, Germany; 6 Institute of Pathology, Klinikum rechts der Isar, Technical University of Munich, School of Medicine, Munich, Germany; 7 Department of General Surgery (Gastrointestinal Surgery), The Affiliated Hospital of Southwest Medical University, Luzhou, Sichuan China; 8 German Cancer Consortium (DKTK), Partner Site Munich, Munich, Germany; 9 CRC 1321 Modelling and Targeting Pancreatic Cancer, Munich, Germany; 10 Department of General Surgery, HPB-Unit, School of Medicine, Acibadem Mehmet Ali Aydinlar University, Istanbul, Turkey

**Keywords:** Cholinergic, Acetylcholinesterase, Pancreatic cancer, Parasympathomimetics, Electroporation

## Abstract

**Background:**

Nerve-cancer interactions are increasingly recognized to be of paramount importance for the emergence and progression of pancreatic cancer (PCa). Here, we investigated the role of *indirect* cholinergic activation on PCa progression through inhibition of acetylcholinesterase (AChE) via clinically available AChE-inhibitors, i.e. physostigmine and pyridostigmine.

**Methods:**

We applied immunohistochemistry, immunoblotting, MTT-viability, invasion, flow-cytometric-cell-cycle-assays, phospho-kinase arrays, multiplex ELISA and xenografted mice to assess the impact of AChE inhibition on PCa cell growth and invasiveness, and tumor-associated inflammation. Survival analyses were performed in a novel genetically-induced, surgically-resectable mouse model of PCa under adjuvant treatment with gemcitabine+/−physostigmine/pyridostigmine (*n* = 30 mice). Human PCa specimens (*n* = 39) were analyzed for the impact of cancer AChE expression on tumor stage and survival.

**Results:**

We discovered a strong expression of AChE in cancer cells of human PCa specimens. Inhibition of this cancer-cell-intrinsic AChE via pyridostigmine and physostigmine, or administration of acetylcholine (ACh), diminished PCa cell viability and invasion in vitro and in vivo via suppression of pERK signaling, and reduced tumor-associated macrophage (TAM) infiltration and serum pro-inflammatory cytokine levels. In the novel genetically-induced, surgically-resectable PCa mouse model, adjuvant co-therapy with AChE blockers had no impact on survival. Accordingly, survival of resected PCa patients did not differ based on tumor AChE expression levels. Patients with higher-stage PCa also exhibited loss of the ACh-synthesizing enzyme, choline-acetyltransferase (ChAT), in their nerves.

**Conclusion:**

For future clinical trials of PCa, *direct* cholinergic stimulation of the muscarinic signaling, rather than *indirect* activation via AChE blockade, may be a more effective strategy.

## Background

The autonomous nervous system has been recently discovered to impact on cancer growth and progression in several solid and hematological cancers [1]. In pancreatic cancer (PCa), surgical denervation via vagotomy or pharmacological suppression of the cholinergic signaling were shown to exert a cancer-promoting effect [2, 3]. In genetically induced LSL-Kras^+/G12D^;Pdx1-Cre (KC) mouse model of PCa, subdiaphragmatic vagotomy led to accelerated cancer growth, and treatment with direct muscarinic agonists restored normal KC phenotype [3]. Here, cholinergic signaling was shown to suppress tumorigenesis and cancer stemness via muscarinic type 1 receptor (M_1_R) signaling [3].

However, activation of muscarinic receptors by its main direct agonist, i.e. acetylcholine (ACh), is a process that is not confined to the autonomous nervous system. In fact, non-neuronal cholinergic signaling is highly common in most cell types and has been shown to regulate basic cell functions, such as proliferation, differentiation and apoptosis [4–6]. In this context, the role of non-neuronal acetylcholine as a local signaling molecule is often disregarded [7, 8]. Together with its corresponding degrading and synthesizing enzymes (acetylcholine esterase/AChE and choline acetyltransferase/ChAT), it is expressed in many eukaryotic cell types and even in plants and primitive uni- and multicellular organisms with no autonomous nervous system [8]. Depending on the muscarinic receptor subtype (M_1_R – M_5_R) to which ACh binds, muscarinic signaling can result in diverse cellular functions. The most potent and relevant ACh receptors that mediate cell proliferation and cell growth are muscarinic receptor type 1 (M_1_R) and type 3 (M_3_R); both widely expressed in most human tissues and especially in gastrointestinal tissues [5, 9]. Local availability of ACh for autocrine and paracrine stimulation of muscarinic receptors regulates not only various physiological cell functions, but has also been shown to critically contribute to tumorigenesis [4]. For instance, in colon, breast and liver cancer, muscarinic receptor activation increased cancer cell proliferation and contributed to cancer progression [10–12]. This effect was mainly attributed to M_3_R signaling [13]. Interestingly, in PCa, M_1_R signaling resulted in reduced tumor growth [3]. This effect was mainly attributed to neuronal cholinergic input, e.g. from the vagus nerve [3]. However, in the PCa microenvironment, there are though several other non-neuronal sources of acetylcholine, such as cancer-associated fibroblasts and pancreatic stellate cells (PSCs), which are thought to influence pancreatic exocrine function via ACh secretion [14].

In this study, we demonstrate that PCa cell growth can also be decelerated by *non-neuronal*, *indirect* cholinergic signaling. This observation suggests that cancer cells, especially pancreatic cancer cells (PCCs), may be largely independent of the autonomous nervous system in their reaction to acetylcholine availability in the tumor microenvironment. Indeed, we demonstrate that human PCCs express high amounts of AChE and that inhibition of non-neuronal AChE suppressed PCC viability and invasion in vitro and in vivo*.* Notably, this effect was induced without surgical vagotomy, but only through non-neuronal, tumor cell intrinsic AChE inhibition. However, survival in a novel genetic, R0-resectable PCa mouse model was not influenced by AChE inhibition in the adjuvant setting. Accordingly the survival of resected PCa patients did not differ based on tumor AChE expression levels. These data imply that direct cholinergic stimulation, rather than indirect activation via AChE blockade, may be a more effective therapeutic strategy in PCa.

## Methods

### Cell culture

The human pancreatic cancer cell lines Panc-1 and SU86.86, the colon carcinoma cell lines SW620 and DLD-1, and the glioblastoma cell line LN229 were purchased from the American Type Culture Collection (ATCC). The T3M-4 cell line was a kind gift by Dr. R. Metzgar (Durham, NC, USA). Cells were kept and cultured in RPMI-1640 supplemented with 10% fetal calf serum (FCS), 100 U/ml penicillin and 100 μg/ml streptomycin (Gibco, Invitrogen, Karlsruhe, Germany) in a 5% CO_2_ humidified atmosphere at 37 °C.

### Matrigel invasion assay

Five thousand PCCs (SU.86.86) were placed into each chemotaxis chamber insert of a 24-well plate (BD Falcon® 8 μm, Heidelberg, Germany) and incubated overnight. After 22 h, the inserts were removed, cleaned of non-migrating cells, fixed in 4% paraformaldehyde, and stained with Vybrant CFDA SE Cell Tracker Kit (Life Technologies, Darmstadt, Germany), and scanned via an automated digital epifluorescence microscope (Keyence BioRevo BZ-9000, Neu-Isenburg, Germany). The number of stained (migrated) cells was counted via ImageJ (version 1.44p, NIH, USA).

### Heterotypic xenograft model

Athymic nude mice (NMRI-Foxn1^nu/nu^) of 4–5 weeks of age and weighing 15-20 g were kept under standard conditions in sterile cages and given food and water ad libitum. Mice were injected subcutaneously (s.c.) in their neck and dorsum with 4 × 10^5^ cells (5000 cells/μl) of the PCa cell line T3M-4, and the animals were divided into two groups for the subsequent treatment: Group I was classified as the “prophylactic” group and treated starting with the day of tumor cell inoculation. Group II were not treated until the 1st week after tumor inoculation to allow the tumor to reach a palpable size. Mice were treated with subcutaneous injections of the AChE inhibitors physostigmine or pyridostigmine as indirect parasympathomimetic agents. Physostigmine, which can cross the blood-brain-barrier (BBB), was administered at 0.1 x LD_50_ and 0.3 x LD_50_, and Pyridostigmine, which cannot cross the BBB, at 0.2 x LD_50_ and 0.4 x LD_50._ Solvent (saline) was injected to the control group. The animals received treatment 5 times a week for a period of 4 and 3 weeks for group I and II respectively. Animals were sacrificed by neck dislocation, and *tumor diameter (mm)* and *local invasive spread* (visible cell spread into neighbouring organs) of cancer cells were assessed.

### R0-resectable, electroporation induced transgenic mouse model of unilocular PCa

Current oncogenic *Kras*-based mouse models of PCa develop multilocular tumors due to constitutive activation of the oncogene in the embryonic phase or due to its inducible activation in the adult age. This modality is not in harmony with human disease, which typically manifests as a single, i.e. unilocular, cancer in the pancreas. To address this discrepancy, Gürlevik et al. recently developed an R0-resectable, electroporation-induced genetic mouse model of unilocular PCa, which is induced upon injection and electroporation of plasmids containing the *Sleeping Beauty* (SB) transposase SB13, a Kras-G12V encoding transposon, and the Cre recombinase into the pancreatic tail of p53floxed mice (*p53*^*fl/fl*^) [15]. Details on the plasmid constructs and the electroporation parameters have been described in the original publication [15]. Upon activation of the Cre recombinase, tumor formation was initiated in a local fashion (the “Pfl” model), and 3 weeks after electroporation, the mice developed a unilocular tumor of the pancreatic tail that is amenable to surgical resection (Fig. 5a-b). The model allows real-life-like performance of neoadjuvant and adjuvant therapy trials with these mice, which exhibit a strongly similar phenotype to the classical, oncogenic Kras-based mouse models of PCa such as *Ptf1a-Cre;LSL-Kras*^*G12D*^ (KC) and *Ptf1a-Cre;LSL-Kras*^*G12D*^*;p53*^*R172H*^ (KPC) [16, 17]. Using this model, we applied adjuvant chemotherapy combining gemcitabine with either physostigmine (at 0.2xLD_50_, i.e. 160 μg/kg, http://datasheets.scbt.com/sc-252784.pdf), or with pyridostigmine (0.2xLD_50_, i.e. 520 μg/kg, http://www.vetpharm.uzh.ch/reloader.htm?wir/00000015/5975_08.htm?wir/00000015/5975_00.htm), applied s.c. three times a week. Gemcitabine was administered as 6 repeats of weekly gemcitabine (100 mg/kg bodyweight diluted in physiological NaCl) intraperitoneally (i.p.), as shown previously [15].

### Multiplex enzyme-linked immunosorbent analysis (ELISA)

Protein levels of IL-6, IL-10 and TNFalpha were measured in mouse serum via the Luminex® MAGPIX® multiplex ELISA system (Merck Millipore, Darmstadt, Germany) according to the instructions of the manufacturer.

### MTT viability assay

To assess human PCa cell line growth, the MTT (3-(4, 5-methylthiazol-2-yl)-2, 5-diphenyl-tetrazolium bromide) assay was used. Cells were seeded at a density of 2000 cells/well in a 96-well plate in serum-free RPMI-1640 medium. Treatment of cells with acetylcholine (Sigma-Aldrich, Taufkirchen, Germany), physostigmine, pyridostigmine or carbachol (all three provided by the internal pharmacy of the Klinikum rechts der Isar) began 12 h after seeding at the concentration of 10, 20, 50, 100 or 300 ng per well (in 100 μl) for physostigmine and pyridostigmine, at 100, 500 and 1000 μM for acetylcholine and at 1 μM, 10 μM, 100 μM, and 1 mM for carbachol. The viability was measured at 0 h, 24 h, 48 h and 72 h after adding the MTT to each well (50 μg/well) and allowed to incubate for 4 h. The formazan products were solubilized with 100 μl of propan-2-ol and the optical density was measured using a photometer at 570 nm.

### Cell cycle analysis

Upon reaching 90% of confluence, T3M-4 cells were treated with ACh at a concentration of 1000 μM, physostigmine and pyridostigmine at 30 ng/μl each and the combined agents at 30 + 30 ng/ μl. The PCC were then harvested, centrifuged at 200×g for 5 min and washed 2 times using phosphate-buffered saline (PBS). They were then resuspended in 1 ml of ice-cold PBS and added dropwise afterwards to ice-cold absolute ethanol for cell fixation. Cells were fixated for 24 h at 4 °C, recentrifuged and resuspended in 500 μl Triton X-100 (Sigma) in PBS with added 100 μg of DNAse-free RNAse A (Sigma) propidium iodide (PI) at a concentration of 20 μg/ml. Cells were then incubated for 15 min at 37 °C and pipetted afterwards into 96-well plates protected from light for data acquisition. Forward and side scatter was measured using a Guava® easyCyte HT Sampling Flow Cytometer.

### Immunoblot analysis

At 90% confluence, PCa cell lines were lysed and 30 μg of protein was separated, electroblotted and the membrane was exposed to monoclonal and polyclonal antibodies (Table 1) at 4 °C overnight. The equal loading of AChE-Blots was assured by re-probing with alpha-Tubulin antibody. The densitometric analysis of the Western Blot was performed via ImageJ (version 1.44p, NIH, USA).

**Table 1:** Primary antibodies. IF: immunofluorescence, WB: Western blot, IHC: immunohistochemistry

Antibody	Clone	Species	Company	Concentration
p44/42 MAPK (Erk1/2)	137F5	Rabbit	Cell Signaling, Leiden, The Netherlands	1:1000
Phospho-p44/42 MAPK (Erk1/2) (Thr202/Tyr204)	D13.14.4E XP®	Rabbit	Cell Signaling, Leiden, The Netherlands	1:1000
Src	36D10	Rabbit	Cell Signaling, Leiden, The Netherlands	1:1000
Phospho-Src (Ser17)	D7F2Q	Rabbit	Cell Signaling, Leiden, The Netherlands	1:1000
AMPKα	D5A2	Rabbit	Cell Signaling, Leiden, The Netherlands	1:1000
Phospho-AMPKα (Thr172)	40H9	Rabbit	Cell Signaling, Leiden, The Netherlands	1:1000
p38α MAPK	7D6	Rabbit	Cell Signaling, Leiden, The Netherlands	1:1000
Phospho-p38 MAPK (Thr180/Tyr182)	D3F9 XP®	Rabbit	Cell Signaling, Leiden, The Netherlands	1:1000
Anti-Acetylcholine-esterase (AChE)		Rabbit pAb	Prestige Antibodies®, Sigma-Aldrich, Taufkirchen. Germany	1:200 (IF) 1:1000 (WB) 1:400 (IHC)
Anti-alpha-Tubulin	Ab11034	Rabbit	Abcam, Cambridge, UK	1:10.000
F4/80	Ab6640	Rat	Abcam, Cambridge, UK	1:75
CD45	Ab10558	Rabbit	Abcam, Cambridge, UK	1:100
ChAT	Polyclonal	Rabbit	Kindly provided by Prof. M. Schemann, TU Munich	1:1000

### Phospho-kinase profiling

The Proteome Profiler Human Phospho-Kinase Array Kit (R&D Systems, Minneapolis, MN, USA) was used to obtain a semiquantitative comparison of the phosphorylation of 43 different human kinases in T3M4 cells that were either treated with 30 ng/μl physostigmine for 5 min or untreated, according to the instructions of the manufacturer.

### Patients and human tissue

Tissue samples from patients undergoing pylorus-preserving Whipple’s procedure for PCa of the pancreatic head were collected and processed as described before [18]. A total of 39 patients were included for the survival analyses (for patient characteristics, please see Table 2). A total of 20 patients were used for the correlation analysis between ChAT content in nerves and Union for International Cancer Control (UICC) stage (median age: 61, 11 male, 9 female, UICC stage distribution: IIA: 4 patients, IIB: 15 patients, III: 1 patient). All patients were informed, and written consent was obtained for tissue collection.

**Table 2:** Patient characteristics. UICC: Union for International Cancer Control

	N
	39
**Sex**	
Male	22 (56.4%)
Female	17 (43.6%)
**Age** (median; min-max)	67.4 (31–83)
**UICC**	
UICC Ia	3 (7.7%)
UICC Ib	8 (20.5%)
UICC IIa	9 (23.1%)
UICC IIb	7 (17.9%)
UICC III	10 (25.7%)
UICC IV	2 (5.1%)

### Immunohistochemistry, immunofluorescence, semiquantitative analysis

Consecutive 3 μm sections from formalin-fixed human tissues were incubated with the corresponding antibodies (Table 1) overnight in a humid chamber at 4 °C. AChE IHC was detected with the Avidin Biotin Complex Peroxidase Standard Staining Kit (Thermo-Fischer, Waltham, USA). Histopathological analysis was performed by two independent observers (PLP, MJ) followed by resolution of any differences by joint review and consultation with a third observer (IED). Scores of 0–3 were given in 0.5 steps according to the amount of immunoreactivity in each tissue samples. For immunofluorescence staining, Alexa® Fluor 488 and 594 antibodies (Invitrogen, Germany, 1:250 concentration) in combination with 4′,6-diamidino-2-phenylindol (DAPI) nuclear stain were utilized.

### AChE activity assay

For comparison of the AChE activity between the human PCa cell lines SU86.86 and T3M4, a colorimetric AChE activity assay kit **(**Sigma-Aldrich, Taufkirchen, Germany), which is based on the Ellman method, was applied according to the instructions of the manufacturer.

### Ethics approval

All animal studies were conducted according to the national regulations and approved by the Regierung von Oberbayern (approval nr. ROB-55.2-2532.Vet_02–16-165 and 55.2-1-54-2531-36-08), and Hannover (15/1949). The study has been approved by the ethics committee of the Technische Universität München, Munich (approval nr. 154/20).

### Statistical analysis

Results are expressed as mean ± SD. Two-group analyses were performed using the unpaired t-test for continuous values and with the Mann–Whitney U test for scores and indices. Linear regression was used for correlating tissue expression of AChE or ChAT with the UICC stages of PCa. Survival analyses were performed with the log-rank test and depicted as Kaplan-Meier curves. All tests were two-sided, and a *p* value < 0.05 was considered to indicate statistical significance. All authors had access to the study data and had reviewed and approved the final manuscript.

## Results

### Pancreatic cancer cells express high amounts of AChE

**Fig. 1:**
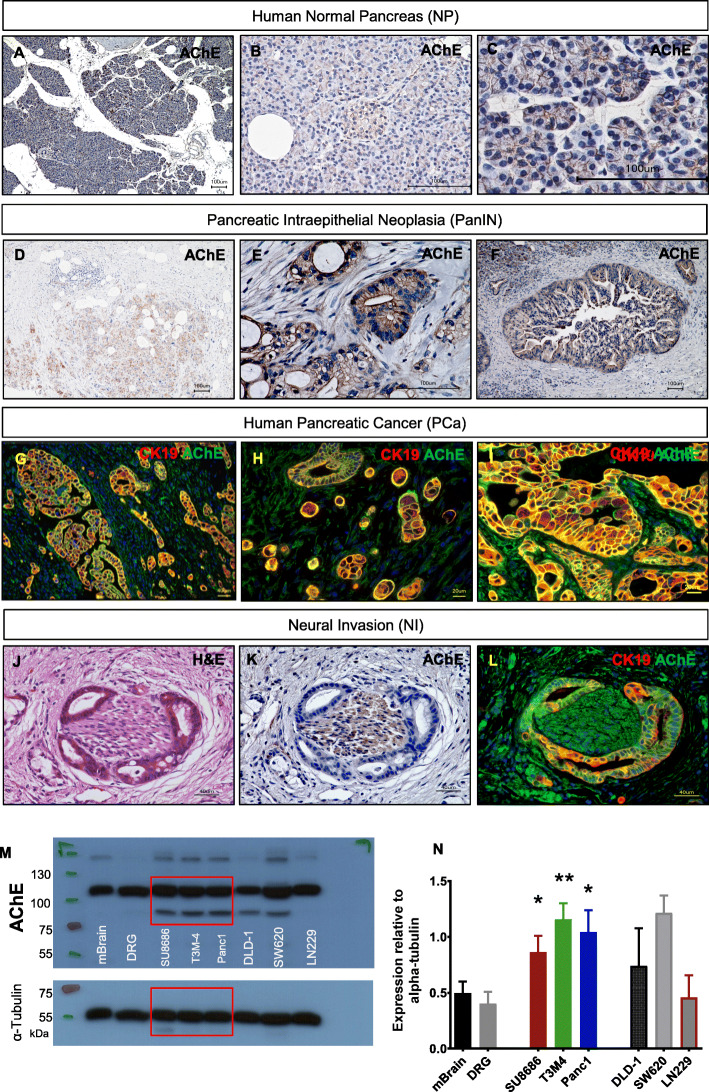
Acetylcholinesterase (AChE) expression in normal pancreas (NP), pancreatic intraepithelial neoplasia (PanIN) and pancreatic cancer (ductal adenocarcinoma/PCa). **a**-**c** Representative immunohistochemical (IHC) photomicrographs of AChE-immunostained normal human pancreas. **d**-**f** Representative images showing IHC staining of AChE in PanIN Grade 3 lesions. **g**-**i** Double-immunofluorescence staining for CK-19, AChE and DAPI in pancreatic cancer. **j**-**l** Representative images of double-immunofluorescence staining for CK-19, AChE and DAPI at sites of (peri)neural invasion in human pancreatic cancer specimens. **m** Immunoblot analysis of AChE (110 kDa & 76 kDa) in mouse brain (mBrain) and mouse dorsal root ganglia (mDRG) mouse as control tissue, in human pancreatic cancer (PCC)-lines (T3M-4, SU.86.86 and Panc-1) as well as colon carcinoma cell lines SW620 and DLD-1 and the glioblastoma cell line LN229. **n** Densitometry graph depicts the quantification of AChE signal relative to alpha-Tubulin content in mDRG, SU.86.86, T3M-4 and Panc1 (*n* = 3 biological replicates), (SU.86.86 *p* = 0.0112; T3M-4 *p* = 0.0018; Panc1 *p* = 0.0074). Results depicted as Mean ± SD. *p < 0.05, **p < 0.01

The role of ACh as a neurotransmitter in the parasympathetic nervous system (PSNS) is well-known, but its non-neuronal function as a local signaling molecule that influences basic cell functions such as apoptosis and proliferation, is often underestimated [9]. The cholinergic system comprising ACh and its synthesizing enzyme, choline acetyltransferase (ChAT), as well as its degrading enzyme, acetylcholinesterase (AChE), have been detected in several cancer entities, such as colon, liver and lung cancer [11, 13, 19]. However, the amount of AChE expression in PCa has not been genuinely analyzed before. By employing immunostaining against AChE, we demonstrated mild to weak staining in normal acinar cells, pancreatic islets and intrapancreatic nerves (Fig. 1a-c). Notably, immunostaining increased in precursor lesions of PCa, i.e. pancreatic intraepithelial neoplasia (PanIN) lesions (Fig. 1d-f). The strongest staining was found in areas of invasive ductal adenocarcinoma with prominent staining localized in the peri- and sub-membranous cell compartment of cancer cells (Fig. 1g-i). Combined staining of AChE and the PCa cell marker Cytokeratin 19 (CK19) using double-immunofluorescence (IF) staining confirmed strong co-localization of CK19 with AChE (Fig. 1g-l), underlining the specificity of AChE expression in cancer cells.

In order to further quantify the expression of AChE in PCa cells (PCC), we isolated dorsal root ganglia (DRG) from postnatal C57BL/6 J mice. DRG neurons incorporate afferent neuronal signals of different qualities and express AChE [20]. Here, immunoblot analysis of commonly used human PCC lines, T3M-4, SU.86.86 and Panc-1, as well as colon carcinoma cell lines SW620 and DLD-1, and the glioblastoma cell line LN229, revealed high levels of AChE expression when compared to DRG of C57BL/6 J mice (Fig. 1m-n). Collectively, these results demonstrate that PCCs express high amounts of AChE in a specific manner.

### AChE inhibition suppresses PCC growth in vitro

**Fig. 2:**
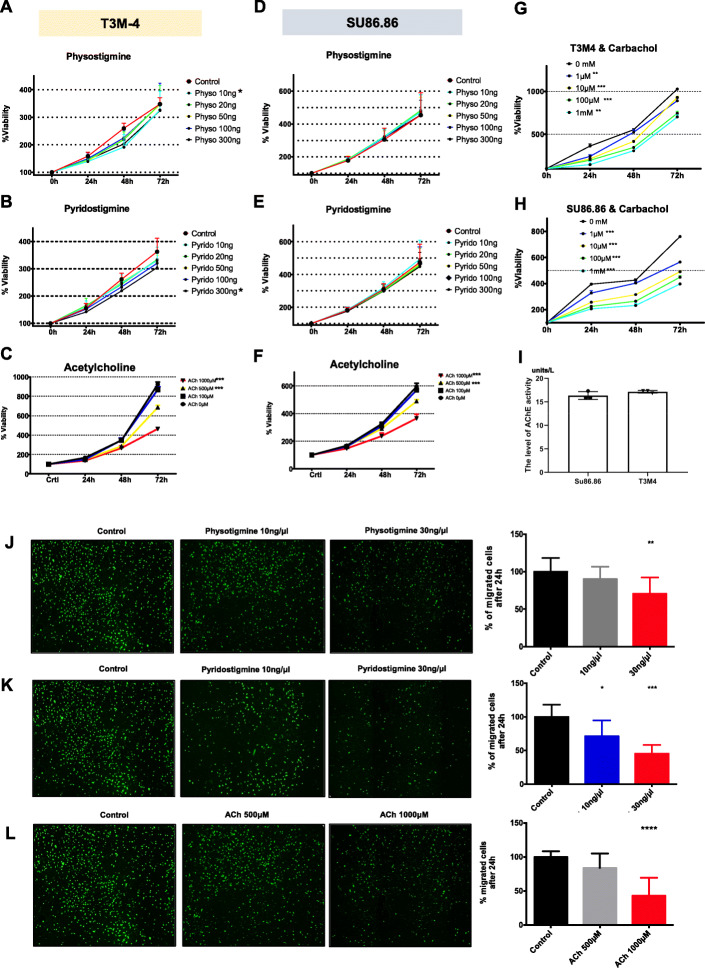
AChE inhibition suppresses PCC proliferation and invasion in vitro*.* **a-h** T3M-4 and SU86.86 human pancreatic cancer cells (PCCs) were treated for 24 h, 48 h and 72 h with indirect parasympathomimetic drugs (physostigmine, pyridostigmine), with the direct parasympathomimetic carbachol or with acetylcholine (ACh) and analyzed for their viability via MTT assay, Graphs shows cell growth of human PCCs over time and area under curve (AUC) values for different treatment regimens (T3M-4: physostigmine/Physo 10 ng: *p = 0.0153; pyridostigmine/Pyrido 300 ng/100 μl:*p = 0.0175; for ACh: T3M-4 and SU86.86: 500 μM ***p < 0.0001; 1000 μM ***p < 0.0001; T3M-4-carbachol: 1 μM: **p = 0.0011, 10 μM: ***p < 0.0001, 100 μM: ***p < 0.0001, 1 mM: **p < 0.0022; SU86.86-carbachol: ***p = 0.0011, unpaired t-test of area under the curve/AUC). **i** AChE activity assay with the SU86.86 and T3M4 cell lines. **j**-**l** Representative photomicrographs of transwell chamber membranes with CFSE–stained SU86.86 PCCs after treatment with physostigmine, pyridostigmine or ACh. Graphs shows the percentage of treated migrated cells compared to solvent-treated treated controls (Physostigmine. 30 ng/μl *p = 0.0102 by unpaired t-test; pyridostigmine: 10 ng/μl *p = 0.0162; 30 ng/μl ****p < 0.0001; ACh: ****p  = 0.0006; by unpaired t-test). All experiments were performed in biological triplicates

Next, we sought to explore the effect of AChE inhibition on cancer cell growth. Administration of physostigmine or pyridostigmine, two commonly used AChE inhibitors, led to significant inhibition of viability in T3M-4, but not in SU86.86 cells (T3M-4-physostigmine: 189 ± 99% with 0.1 ng/μl vs. 217 ± 110% with solvent with 0.1 ng/μl; T3M-4-pyridostigmine: 191 ± 90% with 3 ng/μl vs. 220 ± 117% with solvent, Fig. 2a-f). Similarly, following administration of acetylcholine (ACh), or of carbachol, a direct muscarinic receptor agonist, a dose-dependent growth reduction was evident for both cell lines (ACh-T3M-4: 242 ± 165% with 1000 μM vs. 384 ± 374% with solvent; ACh-SU86.86: 212 ± 116% ng/μl with 1000 μM vs. 297 ± 220% with solvent; Carbachol-T3M-4: 348 ± 285% with 100 μM vs. 511 ± 391% with solvent, Carbachol-SU86.86: 260 ± 145% with 100 μM vs. 421 ± 270% with solvent, Fig. 2c & f-h). To exclude a difference in the basal AChE activity of the cell lines SU86.86 and T3M4, we performed a colorimetric AChE activity assay based on the Ellman method, which revealed no difference in the basal AChE activity of the two cell lines (Fig. 2i). These results confirmed that non-neuronal cholinergic signaling decelerates PCC growth in vitro, which was achieved through increasing ACh availability, through AChE inhibition, or through direct muscarinic stimulation.

### Cholinergic activation inhibits PCC invasion in vitro and in vivo

In order to test the effect of AChE inhibition in PCCs on their invasive potential, we performed Matrigel-based invasion assays with SU86.86 cells (Fig. 2j-l). Also here, in a dose dependent manner, physostigmine inhibited invasion when applied at the concentration of 30 ng/μL (70.4 ± 21.6% of control, Fig. 2i). Pyridostigmine inhibited PCC invasion at intermediate and high concentrations of 10 ng/μL (71.4 ± 23.4% of control) and 30 ng/μL (45.5 ± 13.0% of control), respectively (Fig. 2k), and ACh limited invasion significantly at the higher concentration of 1000 μM (43.2 ± 26.6% of control, Fig. 2l). Collectively, these findings indicated that both AChE inhibition and direct cholinergic activation through ACh inhibit PCa cell invasion in vitro.

**Fig. 3:**
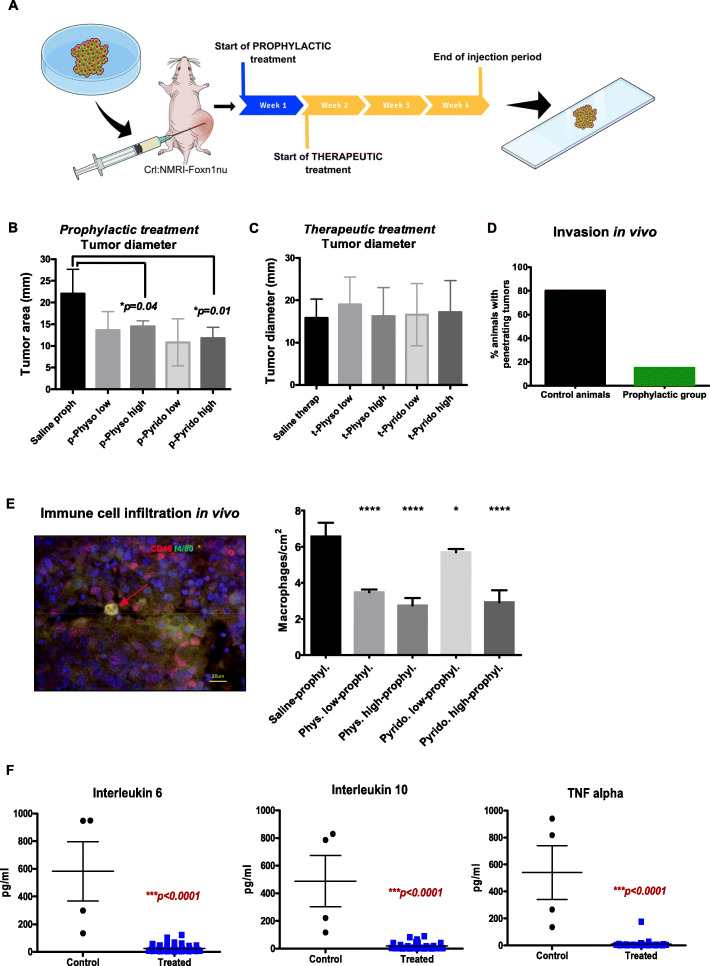
Acetylcholinesterase (AChE) inhibition attenuates the formation of xenografted PCa in mice and decreases tumor-associated inflammation. **a** In vivo xenograft model: human T3M-4 pancreatic cancer cells were transplanted subcutaneously into Crl:NMRI-^Foxn1nu/nu^ mice that were treated prophylactically or therapeutically, the latter beginning at 1 week after tumor induction. Physostigmine and pyridostigmine were administered daily s.c. at 0.1 x LD_50_ (low dose) or 0.3 x LD_50_ (high dose), and Pyridostigmine at 0.2 x LD_50_ (low dose) and 0.4 x LD_50_ (low dose)or saline injection (control). **b** Prophylactic treatment with physostigmine or pyridostigmine resulted in a reduction of the xenografted tumor mass (means ± SD). **c** Therapeutic treatment with AChE inhibitors in mice xenografted with T3M-4 cells. **d** show the percentage of animals that had invasive penetrating tumor growth (Control animals 8/10 = 80% and prophylactically treated animals 6/40 = 15%) **e** Representative IF photomicrograph of double-positive tumor-associated macrophages (TAMs) stained with CD45, f4/80 and DAPI. Graph shows the amount of double-positive cells per square centimeters of tumor tissue compared to the untreated animals (****p < 0.0001 for Phys. low-prophyl., Phys. high-prophyl., and Pyrido. high prophyl. Respectively; Pyrido. low-prophyl. *p = 0.0370 by unpaired t-test). Means ± SEM. * *p* < 0.05; *** *p* < 0.001; **** *p* < 0.0001). **f** Comparative analysis of cytokine levels in the serum of physostigmine or pyridostigmine treated, xenografted mice (“treated”) in comparison with control/saline treated mice (“control”). The results of prophylactically and therapeutically treated animals have been pooled in the graphs

In order to test whether indirect cholinergic activation also reduces pancreatic cancer growth in vivo in animals with intact vagal innervation, we utilized a xenograft mouse model, which was generated by injecting PCC into Crl:NMRI-Foxn1 ^nu/nu^ nude mice. The effect of AChE inhibition was investigated in a prophylactic and a therapeutic treatment group (Fig. 3a). Both groups received daily subcutaneous injections of low- or high-dose physostigmine or pyridostigmine. The prophylactic treatment arm started simultaneously with tumor induction, whereas therapeutic treatment started 1 week after tumor induction. After a 4-week injection period, tumor size was assessed. A significant decrease in the tumor size was observed in animals that received physostigmine or pyridostigmine prophylactically (p) in a dose-dependent manner (Control /saline = 22.0 ± 5.7 mm vs. p-physo-high = 14.5 ± 1.3 mm vs. p-pyrido-high = 11.8 ± 2.5 mm, Fig. 3b). However, therapeutic administration of these indirect parasympathomimetics to established xenograft tumors did not influence tumor size over the course of the treatment (Fig. 3c). In order to evaluate the invasive potential of PCC in vivo, we assessed the proportion of xenografted mice that showed penetrating tumor growth into neighboring organs, i.e. kidneys and lungs, following therapeutic or prophylactic treatment with AChE-blockers. In animals in which AChE was blocked prophylactically, only 15% of the specimens showed penetrating tumor growth, whereas 80% of the control group showed tumor infiltration into neighboring organs (Fig. 3d, Supplementary figure 1). These findings suggested a partially tumor-suppressing effect of non-neuronal cholinergic activation in vivo, yet only in the context of developing tumors.

### Indirect parasympathomimetic agents suppresses immune cell infiltration by tumor-associated macrophages (TAM) and reduce serum cytokine levels in xenografted PCa mice

Non-neuronal cholinergic signaling is also involved in the regulation of the immune system as most immune cells express ACh, AChE, and muscarinic receptors [21]. In this context, we aimed to analyze if indirect cholinergic activation not only has a direct cancer-suppressive effect, but also modulates the immune response in the tumor microenvironment. Therefore, we quantified tumor-associated macrophage (TAM) amounts in pancreatic tumors of the xenograft mouse model. Tumor-associated macrophages are a subpopulation of cytokine-secreting monocytes and have been implicated in playing an important role in the tumor microenvironment (TME). Upon activation, TAM differentiate into M1 or M2 polarized macrophages and release abundant cytokines [22]. Here, we performed double-IF for the murine macrophage marker f4/80 and CD45 (Fig. 3e). Our analysis demonstrated a reduction of CD45^+^/f4/80^+^ − TAM infiltration in murine tumors of physostigmine or pyridostigmine treatment groups (Saline-prophl.: 6.6 ± 0.8 cells/cm^2^, Physostigmine-high: 2.7 ± 0.4 cells/cm^2^, Pyridostigmine-high: 2.9 ± 0.7 cells/cm^2^, Fig. 3e). As cholinergic activation is known to exert a systemic anti-inflammatory effect (“the cholinergic anti-inflammatory pathway”), we then assessed the serum levels of the cytokines interleukin 6 (IL6), interleukin 10 (IL10) and tumor necrosis factor-alpha (TNFalpha) in the xenografted mice (Fig. 3f). Here, we detected a massive suppression of the levels of all these cytokines in all treated groups, regardless of the dosage of treatment, when compared to saline-treated controls (IL6 control: 582.8 ± 428.0 pg/ml, IL6 treated: 25.4 ± 21.5 pg/ml; IL10 control: 488.2 ± 371.6 pg/ml, IL10 treated: 19.4 ± 26.1 pg/ml; TNFalpha control: 540.2 ± 398.4 pg/ml, TNFalpha treated: 10.4 ± 29.3 pg/ml, Fig. 3f). Collectively, these data suggested a prominent suppression of tumor-associated local and systemic inflammation markers in the xenografted PCa mice upon physostigmine or pyridostigmine treatment.

### Cholinergic activation leads to intracellular p-ERK1/2 and p-p38 MAPK inhibition and induces cell cycle arrest

**Fig. 4:**
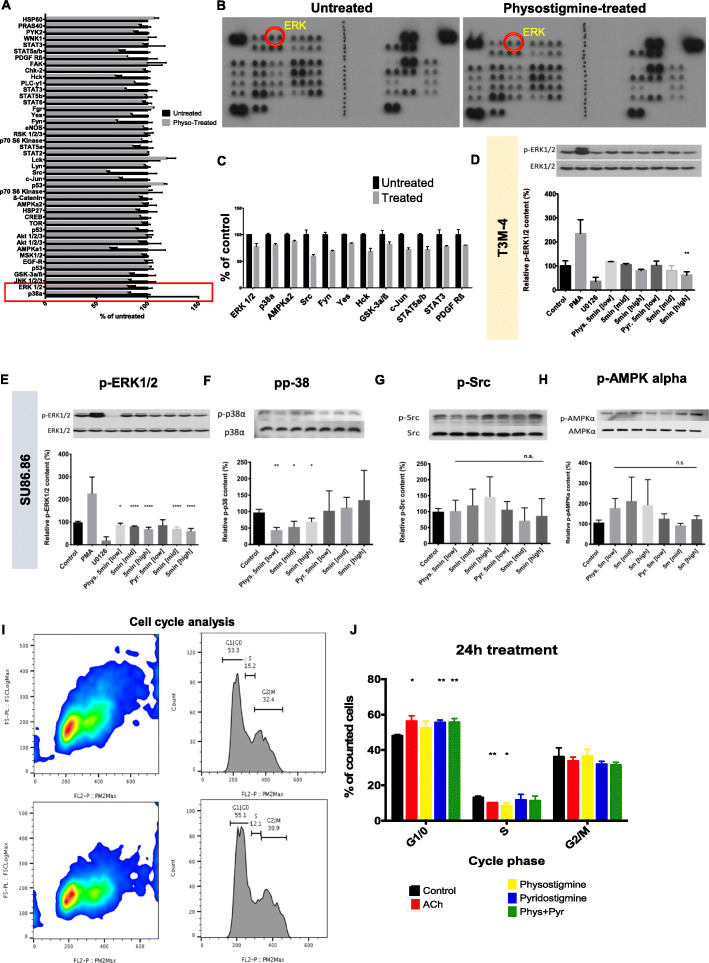
AChE inhibition suppresses ERK & p38 phosphorylation and inhibits cell cycle progression. **a** Bar chart depiction of differences in the signal intensity of various kinases from the human phosphokinase screen in T3M4 cells treated with physostigmine (physo) vs untreated controls. **b**-**c** Representative dot blots and selected bar graphs from the human phosphokinase screen in T3M4 cells treated with physostigmine (physo) vs untreated controls. **d** Western Blot analysis of phosphorylated ERK (pERK)/ERK in T3M-4 cells after 5 min-treatment with phorbol 12-myristate 13-acetate (PMA, an ERK-activator/positive control, Sigma-Aldrich, Taufkirchen, Germany), U0126 (a MEK-inhibitor/negative control, Sigma-Aldrich, Taufkirchen, Germany), physostigmine/Phys and pyridostigmine/Pyr at low, middle and high concentrations (1 ng/μl, 10 ng/μl and 30 ng/μl, respectively). Graph shows quantification of the densitometry of immunoblot for phosphorylated ERK (pERK)/ERK shown in percent of expression compared to control (Pyr. 5 min [high] **p = 0.004 by unpaired t-test). **e**-**h** Western Blot analysis of phosphorylated ERK (pERK)/ERK, of phosphorylated p38 (pp38)/p38, phosphorylated Src (pSrc)/Src and phosphorylated AMPKα (pAMPKα)/AMPKα in SU86.86 cells after 5 min-treatment with phorbol 12-myristate 13-acetate (PMA, an ERK-activator/positive control, Sigma-Aldrich, Taufkirchen, Germany), U0126 (a MEK-inhibitor/negative control, Sigma-Aldrich, Taufkirchen, Germany), physostigmine/Phys and pyridostigmine/Pyr at low, middle and high concentrations (1 ng/μl, 10 ng/μl and 30 ng/μl, respectively). Graphs shows quantification of the densitometry of immunoblots (pERK: Phys. 5 min [low] *p = 0.0109; Phys. 5 min [mid], Phys 5 min [high], Pyr. 5 min [mid] and Pyr. 5 min [high] ****p < 0.0001 by unpaired t-test; pp38: Phys. 5 min [low] **p = 0.0019; Phys. 5 min [mid] *p = 0.0189; Phys. 5 min [high] *p = 0.0302 by unpaired t-test). n.s.: not significant. **i** Scatter plot of propidium iodide (PI) signal in T3M4 PCC untreated (upper graph) and treated (lower graph) with 30 ng/μl of physostigmine and their corresponding cell count plot. **j** shows cell count distribution throughout G1/0-, S- and G2/M-phases of T3M-4 cells. G1/0: ACh 1000 μM *p = 0.0122, Pyridostigmine 30 ng/μl **p = 0.0017, Phys. + Pyr. 15 ng/μl + 15 ng/μl **p = 0.0046; **S:** ACh 1000 μM **p = 0.0033, Physostigmine 30 ng/μl *p = 0.0139 by unpaired t-test). Mean ± SD. *p < 0.05, **p < 0.01, ****p < 0.0001

In order to determine molecular mechanisms responsible for growth and invasion inhibition in PCC upon AChE inhibition, we performed a phospho-kinase antibody array for screening that enables the profiling of 43 different human kinases in two experimental arms (Fig. 4a-b). Here, we compared intensity of phosphorylation of these multiple kinases in T3M4 cells treated with either physostigmine or left untreated, and used the clues from this initial screen for subsequent validation analyses. Among well described mitogen-activated protein kinases (MAPK) that are known to be widely expressed in PCa and involved in cell proliferation, invasion, cell-survival and cell cycling, we found extracellularly regulated kinase 1 and 2 (ERK1/2), p38, proto-oncogene tyrosine-kinase Src (Src) and 5′-AMP-activated protein kinase α (AMPKα) to be altered under the treatment (Fig. 4a-c) [23]. In validation immunoblots with T3M-4 cells, we confirmed the decrease in phosphorylated ERK1/2 (pERK) levels, particularly after treatment with high-dose pyridostigmine (61.5 ± 13.9% of control). This effect was more pronounced for SU86.86 cells, which, after treatment with physostigmine or pyridostigmine, exhibited even more obviously diminished pERK1/2 levels in a dose-dependent manner at both mid-level and high concentrations (physostigmine-mid: 80.5 ± 2.6% of control, physostigmine-high: 69.2 ± 7.9% of control, pyridostigmine-mid: 70.2 ± 8.2% of control, pyridostigmine-high: 60.3 ± 11.8% of control, Fig. 4e). As an essential component of the MAPK signal transduction pathway, p38 reacts to extracellular stimuli and mediates cellular responses [24, 25]. In our experiments, phosphorylation of p38 was abolished upon administration of low (43.7 ± 8.2% of control), mid- (53.4 ± 17.3% of control) and high (69.3 ± 10.9% of control) physostigmine concentrations, but not via pyridostigmine (Fig. 4f). However, following treatment with either of these drugs, there was no significant change in the amount of intracellular p-Src nor p-AMPKα (Fig. 4g-h). In summary, our experiments demonstrated that inhibition AChE reduced ERK phosphorylation.

To investigate whether cell cycle progression of PCC is also affected by AChE inhibition, we performed a propidium iodide-(PI-) based, flow cytometric cell cycle analysis (Fig. 4i-j). After administering ACh, significantly more PCCs were observed in the G1/0 phase (ACh: 56.1 ± 3.2% vs. Control: 47.8 ± 0.9%) and fewer cells in the S-phase (ACh: 10.0 ± 0.1% vs. Control: 13.0 ± 0.8%, Fig. 4i-j). Physostigmine did not alter G1/0-phase amount, but reduced S-Phase cell-count (8.4 ± 1.7%), and pyridostigmine enhanced G1/0-phase count (55.3 ± 1.5%), but did not alter the S-Phase cell count (Fig. 4i-j). No significant difference in cell count was noted for cells in G2/M-Phases, however following all treatments a trend towards lower cell counts was observed. Overall, we thus detected a cell cycle arrest in G1/0 phase following AChE inhibition.

### Adjuvant indirect cholinergic treatment does not impact survival in a resectable PCa mouse model

**Fig. 5:**
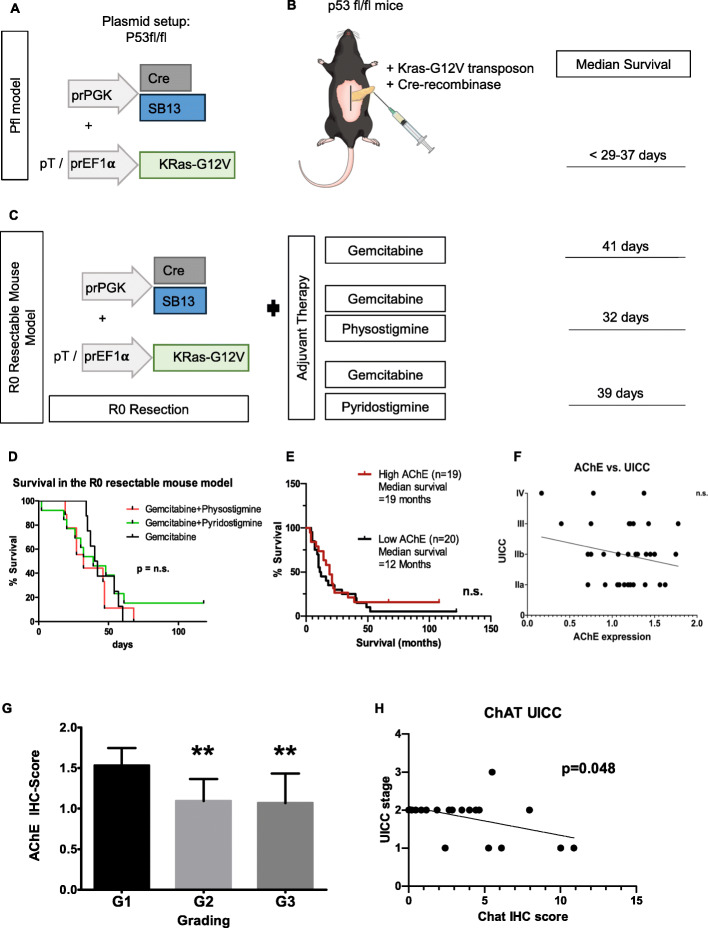
Clinical impact of AChE in the R0 resectable, transgenic pancreatic cancer mouse model and in human PCa. **a**-**b** Plasmids containing the *Sleeping Beauty* (SB) transposase SB13, a Kras-G12V encoding transposon, and the Cre recombinase were injected and electroporated into the pancreatic tail of p53floxed mice (*p53*^*fl/fl*^). (Pfl model). For details on plasmid constructs, please refer to [15]. **c**-**d** After pancreatectomy and adjuvant chemotherapy with gemcitabine, mice of the Pfl-genotype exhibited a median survival of 41 days. Combinational therapy of pancreatectomy with adjuvant gemcitabine and physostigmin led to a median survival of 32 days, and with adjuvant gemcitabine and pyridostigmine to 39 days (n.s.: not significant). **e** Survival rate of PCa patients with high (*n* = 19) and low (*n* = 20) AChE presence based on median immunohistochemistry/IHC-Score (n.s., log-rank test.). Kaplan-Meier analysis did not reveal a significant difference in survival between both groups. **f** Correlation analysis (linear regression) of cancer tissue AChE expression based on semiquantitative IHC score and UICC tumor stage (n.s.: not significant). **g** Correlation of semiquantitative immunohistochemistry (IHC) scores for AChE expression and tumor grading (G1-G3). (G1 vs. G2 **p = 0.018; G1 vs. G3 **p = 0.015; Mann-Whitney U test). **h** Correlation analysis (linear regression) of ChAT expression within nerves of human PCa tissues based on semiquantitative IHC score and UICC tumor stage. Graph shows a negative correlation between ChAT expression and tumor stage, indicating that low ChAT expression is correlated to higher tumor stages, while high ChAT expression correlates to low tumor stages (r^2^ = 0.1988, *p* = 0.048)

In order to translate our findings into a clinically relevant setting, we used a novel R0-resectable, genetically induced PCa mouse model [15]. In this model, plasmids containing the *Sleeping Beauty* (SB) transposase SB13, a Kras-G12V encoding transposon, and the Cre recombinase were injected and electroporated into the pancreatic tail of p53floxed mice (*p53*^*fl/fl*^) via mini-laparotomy [15]. Upon activation of the Cre recombinase, tumor formation was initiated in a local fashion (the Pfl model), which is in contrast with the multilocular tumor growth of classical genetically induced mouse models of PCa (Fig. 5a-b). Three weeks after the tumor induction, the animals developed macroscopically visible tumors. After pancreatic tail resection, mice received adjuvant chemotherapy with gemcitabine. Here, mice of the Pfl-genotype exhibited a median survival of 41 days (Fig. 5c). When adjuvant gemcitabine treatment was combined with physostigmine, median survival was 32 days. Combinational therapy with gemcitabine and pyridostigmine was associated with a median survival to 39 days (Fig. 5c-d). The most common reason for death was combined local and distant (hepatic or peritoneal) recurrence. Thus, the AChE inhibitors did not generate any additive survival benefit in this innovative, adjuvant therapy setting.

### Correlation of tissue AChE and ChAT expression with clinicopathological variables in human PCa

Lastly, to compare our findings from this translational mouse model to human PCa, we analyzed the expression patterns of AChE and the ACh synthesizing enzyme ChAT in human PCa tissues (*n* = 39) by semiquantitive immunostaining scores and correlated to clinicopathological variables of the corresponding patients. Furthermore, high vs. low scores of AChE immunostaining (separated by the median score) did not result in any difference in the overall survival rate of the PCa patients (Fig. 5e). Accordingly, tissue expression scores of AChE did not associate with different UICC tumor stages (Fig. 5f). Interestingly, higher tumor grades, i.e. a poor tumor differentiation, were associated with significantly lower AChE IHC scores (G1: 1.5 ± 0.2, G2: 1.1 ± 0.3, G3: 1.0 ± 0.4, Fig. 5g), suggesting a spontaneous loss of AChE in increasingly aggressive PCa. This was surprising, as we originally hypothesized that high AChE expression, i.e. diminished cholinergic input, would be associated with worse survival and poor differentiation. As this was not the case, we quantified the expression level of choline acetyltransferase enzyme (ChAT), which catalyzes the formation of acetylcholine, in the nerves of these tissues via immunohistochemical scoring. Correlation of ChAT expression levels to tumor stage revealed that high expression levels ChAT were indeed associated with low tumor stages (r^2^: 0.20, *p* = 0.049, Fig. 5h). Hence, we concluded that advanced tumor stages were characterized by low cholinergic input due to low ChAT expression, and yet also by suppression of the degrading enzyme AChE. This simultaneous suppression of the ACh-synthesizing and ACh-degrading intrinsic mechanisms in PCa may explain the lack of a prognostic effect of tumor AChE levels in established mouse and human PCa.

## Discussion

The present study suggested an anti-proliferative and anti-invasive effect of non-neuronal cholinergic signaling in pancreatic cancer. Inhibition of endogenous, non-neuronal AChE decelerated PCC growth and invasiveness in vitro & in vivo, which was linked to intracellularly reduced MAPK phosphorylation and reduced downstream phosphorylation of ERK1/2 and p38. Furthermore, prophylactic cholinergic activation in PCa mouse models with intact vagal innervation reduced both tumor invasiveness in vivo and immune cell infiltration by tumor-associated macrophages. However, administration of parasympathomimetic agents as co-adjuvant therapy together with gemcitabine did not influence the overall survival of mice in a resectable, transgenic mouse model of unilocular genetic PCa. Accordingly, AChE did not correlate to survival in human PCa and was actually suppressed in parallel with ChAT in higher grade tumors. Therefore, our study suggests that for targeting PCa, *direct* cholinergic stimulation of the muscarinic signaling, rather than *indirect* activation via AChE blockade, may be a more effective therapeutic strategy.

Various studies have previously reported a cancer-promoting effect of the vagus nerve. In a mouse model of gastric cancer, surgical vagotomy decreased gastric mucosal thickness and cellular proliferation [26, 27]. The effect was thought to be mediated via muscarinic receptor type 3 (M_3_R) signaling, since knock-out of the M_3_R suppressed gastric cancer. These studies led to the conclusion that vagal innervation promotes gastric cancer via muscarinic M3 receptor in a Wnt mediated pathway [28]. However, in PCa, Renz et al. demonstrated that ablation of the vagal nerve actually accelerated cancer progression [3]. Treatment with the muscarinic receptor agonist, bethanechol was able to reverse the accelerated cancer progression due to vagal ablation. Overall, this study along with others led to the general hypothesis that vagal innervation has a cancer-attenuating effect in the pancreas. In a wide-scale analysis of neural fiber quality in PCa specimens, we previously found a low parasympathetic fiber content of nerves that were invaded by pancreatic cancer cells [29]. In line with these previous studies, in the current study, we were able to demonstrate a cancer-cell-suppressive effect of AChE inhibition and thus indirect cholinergic activation in vitro and in vivo. However, this effect was obtained without directly interfering with the autonomous nervous system, and yet did also not translate into an improved clinical outcome, i.e. survival, in mouse PCa. These findings are of major importance for all studies related the role of cholinergic / parasympathetic nervous system in cancer, since all components of the cholinergic system (ACh, acetylcholinesterase, muscarinic acetylcholine receptors, acetylcholine transferase) are not exclusively expressed by neurons but ubiquitously present in almost all mammalian cells, including non-neuronal cells [8].

In order to understand non-neuronal cholinergic signaling and its involvement in basic cellular functions, such as proliferation and differentiation [6], one has to consider the different subtypes of muscarinic receptors that initiate these diverse cellular outcomes. There are 5 different muscarinic receptor subtypes (M1R – M5R), all of which are G protein-coupled receptors but may lead to different intracellular cascades in order to exert different extracellular outcomes. Upon activation, odd-numbered muscarinic receptors couple to G proteins that activate phospholipase C-β to initiate the phosphatidylinositol trisphosphate cascade, whereas even numbered muscarinic receptors couple to G proteins that inhibit adenylyl cyclase activity [9]. This complexity explains in part why, for instance, activation of the M_3_R subtype has been shown to promote cancer cell proliferation in gastric cancer, whereas activation of M_1_R subtype has been shown to attenuated pancreatic cancer proliferation. Although the role of muscarinic receptors in colon cancer has been previously characterized, most studies only focused on one of the 5 different receptors [9, 13]. Even though there has been extensive research about the tissue-specific expression of muscarinic receptors, a comprehensive overview about the role of muscarinic receptors and its ligands in different cancer entities is still missing.

The lack of basic research on non-neuronal cholinergic signaling is even more evident for PCa. Very little is known about the role of the different muscarinic receptor subtypes as well as other components of the cholinergic-signaling-machinery, such as ChAT, AChE or ACh expression in PCa.

Therefore, our study contributes to the attempts to understand the non-neuronal AChE in PCa. Here, we demonstrated mild to weak staining in premalignant lesions, with increasing staining in overt pancreatic cancer. Mammals express 3 different classes of AChE, which differ with regard to their subunits. Each type of AChE has a different 3′ RNA sequence with a corresponding C-terminal sequence, which encodes the respective subunit. The AChE _H_ subunit contains a hydrophobic C-terminal sequence forming amphilic monomers and dimers and incorporates a GPI [6]. It is therefore often found closely spaced to the cell membrane. This would explain the perimembranous staining found in our study.

Based on our findings, *indirect* activation of cholinergic signaling via AChE inhibition is not sufficient to achieve a survival benefit in PCa, although it resulted in a prominent suppression of the tumor-associated inflammation in the tumor, and a drop of serum cytokine levels. This conclusion, which is based on our findings from a translational mouse PCa model and from human PCa, underlines that increasing the cholinergic input for attenuating PCa progression and for improving patient survival will probably not be possible via administration of two widely used clinical drugs, i.e. physostigmine and pyridostigmine. In contrast, Renz et al. made use of a direct activator of muscarinic cholinergic signaling. i.e. the bethanechol, which did result in improved survival in the KPC model of PCa [3]. In the present study, we combined the indirect cholinergics with an older chemotherapeutic, i.e. gemcitabine, in the adjuvant and palliative treatment. It is imaginable that a combination with a more current regimen such as gemcitabine and nab-paclitaxel or with FOLFIRINOX may yield even more potent results. Nonetheless, we observed immunosuppressive effects of indirect cholinergic stimulation in our study. It is conceivable that in a more humanized model, this immunmodulatory effect of indirect cholinergic stimulation may have been much greater. The NMRI-Foxn1^nu/nu^ model is deficient with regard to T cell function due to a thymus abnormality. For this reason, we primarily assessed macrophage distribution upon treatment with indirect cholinergic agents. As a recent report showed, cholinergic activity of the vagus nerve inhibits macrophage-derived tumor necrosis factor-α secretion via T-cell derived acetylcholine in the spleen [30]. Our data suggest that there may also be a T-cell-independent, immunosuppressive effect of cholinergic activation on macrophages.

## Conclusion

The present study suggests that the growth-suppressive effects of inhibition of the non-neuronal, cancer-cell-intrinsic AChE via indirect parasympahomimetic drugs do not translate into improved survival in PCa. Therefore, targeting PCa over its nervous-cholinergic side should pursue the track of direct, rather than indirect, parasympathetic-cholinergic activation. Future clinical study designs should thus include novel, *selective direct* muscarinic agonists, rather than clinically available, widely used indirect agonists.

## Supplementary Information

**Additional file 1.**

## Data Availability

All data are available from the Authors upon reasonable request.

## Abbreviations

ACh: Acetylcholine
AChE: Acetylcholine esterase
ChAT: Choline acetyltransferase
ERK: Extracellular signal-regulated kinase
MAPK: Mitogen-activated protein kinase
M_1_R: Muscarinic receptor type
M_3_R: Muscarinic receptor type
PCa: Pancreatic cancer
PCC: Pancreatic cancer cells
