# Supplementary figures and images for: Indirect cholinergic activation slows down pancreatic cancer growth and tumor-associated inflammation
Source: J Exp Clin Cancer Res. 2020 Dec 24;39:289. doi: 10.1186/s13046-020-01796-4 (PMC7758936; doi:10.1186/s13046-020-01796-4)

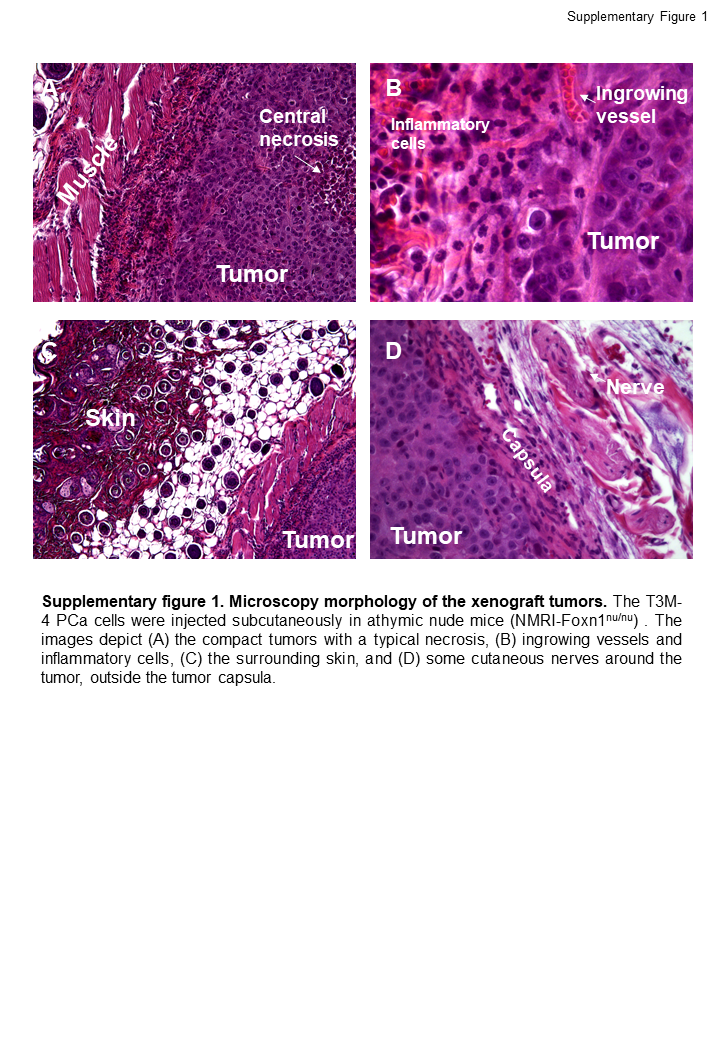

Supplement: Supplementary file 1 — Additional file 1. [file 13046_2020_1796_MOESM1_ESM.tif]
